# Veterinary Department, University of Pennsylvania

**Published:** 1895-08

**Authors:** 


### AMONG THE COLLEGES.

### Veterinary Department, University of Pennsylvania.

The reunion of the graduates of the Veterinary Department, University of Pennsylvania, brought together some forty of her alumni, among them many of the earlier classes, representing New Jersey, Delaware, Pennsylvania, and District of Columbia. Almost all the Veterinary Faculty were present, including Dr. John Marshall, Dean. The task of toast-master was well performed by Dr. A. E. Conrow, of New Jersey, who called, among many others, the following toasts: "The Veterinary Department," responded to by Dean Dr. John Marshall; "Faculty," Professor Harger; "Veterinary Profession," Professor Pearson; "Veterinary Ethics," Professor Adams; "Alumni," Dr. W. H.

Ridge; "The Infants," by Dr. Black, of the graduating class. A thoroughly enjoyable time was afforded all who were privileged to be present.

---

